# ISA-TAB-Nano: A Specification for Sharing Nanomaterial Research Data in Spreadsheet-based Format

**DOI:** 10.1186/1472-6750-13-2

**Published:** 2013-01-14

**Authors:** Dennis G Thomas, Sharon Gaheen, Stacey L Harper, Martin Fritts, Fred Klaessig, Elizabeth Hahn-Dantona, David Paik, Sue Pan, Grace A Stafford, Elaine T Freund, Juli D Klemm, Nathan A Baker

**Affiliations:** 1 Knowledge Discovery and Informatics, Pacific Northwest National Laboratory, Richland, WA 99352, USA; 2 SAIC-Frederick, Inc, Frederick National Laboratory for Cancer Research, Information Systems Program, Rockville, MD 20852, USA; 3 Department of Environmental and Molecular Toxicology, School of Chemical, Biological and Environmental Engineering, Oregon State University, Corvallis, OR 97331, USA; 4 Pennsylvania Bio Nano Systems, LLC, Doylestown, PA, USA; 5 Lockheed Martin, Rockville, MD 20852, USA; 6 Department of Radiology, Stanford University, Stanford, CA 94305, USA; 7 The Jackson Laboratory, Bar Harbor, Maine 04609, USA; 8 3rd Millennium, Inc, North Smithfield, RI 02896, USA; 9 Center for Biomedical Informatics and Information Technology, National Cancer Institute, Rockville, MD 20852, USA; 10 Knowledge Discovery and Informatics, Pacific Northwest National Laboratory, PO Box 999, MSID K7-28, Richland, WA 99352, USA

## Abstract

**Background and motivation:**

The high-throughput genomics communities have been successfully using standardized spreadsheet-based formats to capture and share data within labs and among public repositories. The nanomedicine community has yet to adopt similar standards to share the diverse and multi-dimensional types of data (including metadata) pertaining to the description and characterization of nanomaterials. Owing to the lack of standardization in representing and sharing nanomaterial data, most of the data currently shared via publications and data resources are incomplete, poorly-integrated, and not suitable for meaningful interpretation and re-use of the data. Specifically, in its current state, data cannot be effectively utilized for the development of predictive models that will inform the rational design of nanomaterials.

**Results:**

We have developed a specification called ISA-TAB-Nano, which comprises four spreadsheet-based file formats for representing and integrating various types of nanomaterial data. Three file formats (Investigation, Study, and Assay files) have been adapted from the established ISA-TAB specification; while the Material file format was developed *de novo* to more readily describe the complexity of nanomaterials and associated small molecules. In this paper, we have discussed the main features of each file format and how to use them for sharing nanomaterial descriptions and assay metadata.

**Conclusion:**

The ISA-TAB-Nano file formats provide a general and flexible framework to record and integrate nanomaterial descriptions, assay data (metadata and endpoint measurements) and protocol information. Like ISA-TAB, ISA-TAB-Nano supports the use of ontology terms to promote standardized descriptions and to facilitate search and integration of the data. The ISA-TAB-Nano specification has been submitted as an ASTM work item to obtain community feedback and to provide a nanotechnology data-sharing standard for public development and adoption.

## Introduction

As we enter the National Nanotechnology Initiative’s second decade [1], the early concerns regarding rapid product development without extensive regulatory review have shifted to today’s modest commercialization rate and pursuit of plausible risk assessments. Responsible development of nanotechnology will be influenced strongly by the public’s perception of both promise and risk coupled with the expectation that there are responsible parties addressing any remaining uncertainty [2,3]. Meeting this expectation requires effective multidisciplinary communication that often involves the exchange of complex datasets. The responsible development of nanotechnology products will be empirically-driven, grounded on effective informatics [4] for a variety of purposes; e.g., discussing ecotoxicity data with a regulator or modeling environmental fate for the purpose of setting appropriate disposal instructions or regulating the design of nanoparticles for an effective biological application.

A cross-cutting need in the nanotechnology community is the development of standards to support both meaningful data submission to public repositories and information exchange among investigators [5]. In order to move toward the rational design of nanomaterials for biomedical and other applications, it is critical to recognize and exploit the nanomaterial physicochemical properties that drive biological behavior. Data mining and computer simulation are essential for deriving this information; however, the datasets needed to support such modeling and computational studies are sparse and stored across a variety of nanotechnology repositories and resources. The lack of standardized data formats and the non-uniformity of information reported have proven to be significant barriers to effective and meaningful data sharing and re-use. We have addressed this problem by developing a spreadsheet-based data exchange format specification, called ISA-TAB-Nano, which provides a general framework for representing and integrating diverse types of data related to the description and characterization of nanomaterials. As noted by Dr. Francis Collins, open access to technologies, tools, and databases has become increasingly critical for accelerating biological discovery [6,7]. Open access databases necessitate open standards for representing the information to be exchanged. A number of nanomaterials resources are now becoming available leading to an opportune moment to introduce an open standard for nanomedicine. In this paper, we discuss the main features of ISA-TAB-Nano file formats and how they can be used to represent and share nanomaterial data.

### Data exchange formats and their uses

The ability to share data meaningfully among investigators from separate biomedical disciplines is a challenge [8-11]. In order to understand and use experimental data generated by another investigator, one must have sufficiently detailed information about how the data were generated, using mutually understood terminology. These requirements can be facilitated through a shared data exchange format that captures information between two laboratories or between investigators and public repositories. Many XML-based and spreadsheet-based data exchange formats have been successfully developed and adopted across the biomedical community; examples include the SBML – Systems Biology Markup Language [12]; BioPAX – Biological Pathway Exchange [13]; MAGE-ML – Microarray and Gene Expression Markup Language [14]; MAGE-TAB – Microarray Gene Expression Tabular [15]; and ISA-TAB – Investigation/Study/Assay Tabular [16-18].

Compared to XML-based formats, spreadsheet-based formats offer the advantage that they can be created, viewed, and edited by the investigator using software (e.g., Excel) that is broadly available and familiar to most researchers. The most widely adopted spreadsheet-based formats of biological data are MAGE-TAB [15] and ISA-TAB [16-18]. MAGE-TAB supports the capture and exchange of microarray data in accordance with the minimal information standards adopted in this field [19]. It was developed in response to the need for a format that could be created and used by laboratories lacking the dedicated bioinformatics personnel necessary to support its predecessor XML-based MAGE-ML format. The ease of use of MAGE-TAB has facilitated its broad adoption across the microarray community and it is supported by several public repositories (ArrayExpress [20], caArray [21], the Stanford Microarray Database [22]) and analysis tools (e.g., GenePattern [23], Bioconductor [24]). In addition, data from The Cancer Genome Atlas Project (TCGA) are made available in MAGE-TAB format. Based on the wide acceptance of MAGE-TAB, a generalization of this format (ISA-TAB) was developed to support the broader community need for a common format to support the exchange of data about ‘omics studies employing a combination of methodologies (e.g., genomics, transcriptomics, proteomics). Multiple public and private repositories are now ISA-TAB compliant [25] and an open-source tool suite has been developed to support the use of this format [16-18].

### The need for a data exchange format to share nanomaterial data

Information describing the chemical composition and three-dimensional (3D) structure of nanomaterials is often represented in an unstructured fashion, with no standardized way to associate this information with the assay data from characterization studies. Nanomaterials are diverse: chemical composition, size, geometry, morphology and surface chemistry all play important roles in determining the biological properties of resulting nanomaterials. Complex nanomaterials are often multi-component systems formulated with small molecules for specific biomedical applications such as drug delivery, imaging, and detection of diseases. Such complex formulations make the representation of exact chemical composition, physical structure, and properties very challenging and very necessary.

Nanomaterial analysis requires knowledge of the characterization experiment details so that the results can be accurately and interpreted. Nanomaterial interactions with biological systems are complex and, accordingly, associated data are often generated at multiple levels of detail. The properties of nanomaterials are analyzed based on the data available from different characterization studies, such as *in vitro* and *in vivo* analysis of biological activities and abiotic studies of physicochemical properties. Each characterization itself involves a wide range of variables using different types of assays, which can also vary depending on the endpoint measured, technology used, and the protocol applied. Furthermore, even minor variations in the chemical composition, assay protocols and conditions, and time point of analysis (the elapsed time since the nanomaterial was synthesized), can give rise to different characterization results. Currently, most of the measurements on nanomaterial properties depend on non-standardized protocols and diverse instrumentation technology types. Experimental results are dependent on protocol details that are not consistently captured or reported [26]. For example, measurements of certain parameters, such as size, are dependent on the instrumentation and the sample preparation method; this information needs to be captured in the form of metadata to fully interpret such measurements.

### ISA-TAB-Nano specification

To address the challenges associated with the sharing and re-use of nanomaterial data, we have developed the ISA-TAB-Nano specification, a standard format for representing and sharing data pertaining to the description and characterization of nanomaterials. ISA-TAB-Nano is intended to facilitate the submission and exchange of assay metadata and nanomaterial descriptions, along with other information (raw/derived data, image data, protocol information), among individual researchers and to/from nanotechnology online resources. We expect that ISA-TAB-Nano will provide organizations with a standard approach to representing nanomaterial data that will enable researchers to share nanomaterial information effectively and to make cross-material comparisons.

## Methods

In 2008, the Nanotechnology Working Group (Nano WG) was established as part of National Cancer Institute (NCI) cancer Biomedical Informatics Grid (caBIG^®^) program, to support the informatics needs of nanomedicine researchers. Today, the group has over 20 active participants who represent a broad range of expertise and come from academic, industrial, government agency and other institutions. Beginning in March 2010, a sub-group of the Nano WG began developing a specification to facilitate the exchange of nanomaterial data amongst research labs and with public repositories of nanoparticle information. An important goal of this effort from the outset was to use and extend concepts from the caBIG^®^ Life Sciences Domain Analysis Model [27] while using the NanoParticle Ontology [28] and other ontologies as terminology sources. Recognizing that nanoparticle assays have many of the same challenges as microarray assays, the Nano WG looked to the microarray community for lessons learned. The ISA-TAB standard [16,18] forms the basis for our ISA-TAB-Nano format because of its flexibility in supporting several different types of assays of interest including physicochemical*, in vitro*, and *in vivo* assays.

The ISA-TAB-Nano file formats were evaluated using several nanomaterial datasets from the cancer Nanotechnology Laboratory Portal (caNanoLab) [29] and individual researchers with applications ranging from biomedicine to nanotoxicology. This diversity allowed for format refinement and enabled support for a broad range of interests. Test case studies were conducted and feedback was provided to the ISA-TAB-Nano development team. Example datasets are available from the ISA-TAB-Nano website at https://wiki.nci.nih.gov/x/HgiG.

### Basis of ISA-TAB-Nano

#### ISA-TAB

The ISA-TAB-Nano specification is based on the ISA-TAB specification [16-18,25]. ISA-TAB defines three file formats for sharing the metadata associated with ‘omics (e.g., proteomics, genomics, metabolomics, and transcriptomics) – based experiments. The three files are called Investigation file, Study file, and Assay file. Non-standard (user-defined) file formats such as image files, spreadsheet files containing raw/derived data, and protocol documents, can also be referenced in appropriate fields within the Investigation, Study, and Assay files. By providing a standard format for representing the metadata related to each ‘omics experiment, the ISA-TAB file formats facilitate the integration and sharing of ‘omics data in a meaningful and standardized way.

The ISA-TAB Study file is used to record information about the source and characteristics of a subject (sample) under study as well as the different treatment protocols that are applied to prepare the samples for the analysis (assay). The subject under study could be an organism, a sample taken out of the organism, or any other sample. The Assay files are used to record the assay metadata along with references to the names (or URIs) of the raw/derived data and image files. In the ISA-TAB framework, the type of assay is determined by the endpoint of measurement and by the technology (technique) used to measure that endpoint. Therefore, a single study (of a given sample) can comprise several types of assays. The Investigation file serves to relate the different types of assays to their corresponding studies. In particular, the Investigation file is used to record reference information about each Study file and Assay file; point of contact; publications; the parameters, instruments, software, and reagents of a protocol; the factors of the study (a factor is defined as any independent variable that is varied in an experiment to investigate its effect on the subject under study) and the terminology sources referred to in the Investigation, Study and Assay files.

### Biospecimens versus material samples

An important extension to ISA-TAB required to support nanomaterials came from the need to distinguish samples derived from biological sources (e.g., from cell lines, tissues, body fluids, etc.) and samples derived from non-biological sources (e.g., nanoparticle formulations, small molecule drug, etc.). Hence, for the ISA-TAB-Nano specification, we consider a sample derived from biological sources as a *biological specimen* or *biospecimen material type*, while referencing samples derived from non-biological sources as *material samples*.

The ISA-TAB Study file can be used to describe the source and characteristics of biological specimens, however, it is not suitable for describing nanomaterials. In this work, we have designed a “Material” file format for describing nanomaterials. In general, this file format is applicable for describing any material sample, nanoscale or otherwise, as discussed later. Hence, in ISA-TAB-Nano, the concept of a sample (as used in ISA-TAB specification) is extended to distinguish between biological specimens and material samples by incorporating information about the latter into the Material file.

### Scope of a ISA-TAB-Nano Material file

The Material file is designed to capture information related to the description of nanomaterials. Typically, the description of a nanomaterial is complex and includes different types of information (name, type, characteristics) related to the whole nanomaterial and its individual chemical components. A typical example is that of a nanoparticle formulation [5,28], which can be generally described as a multi-component system containing chemical components that may be distinguished as nanoparticles, chemical constituents of the nanoparticle, functionalizing agents, and components of the medium in which the nanoparticles may be suspended. These chemical components can be described and identified based on their molecular structure, biochemical role, and function [28]. The types of information needed to describe nanoparticles and their formulations include specification of the linkages existing between the different components of the formulation (e.g., encapsulation, entrapment, amide linkages, etc.); the physical state of the formulation (e.g., emulsion, solid, hydrogel, etc.); the properties (physical, chemical, and functional) of each chemical component; the intended functions and applications of the nanoparticle; the nanoparticle design principles (or rationale); and the type of stimulus that may be required for activating a specific function of the nanoparticle (e.g., magnetic field, ultrasound, pH change, etc.) [28]. All of these types of information were taken into account when developing the specification for the ISA-TAB-Nano Material file format.

## Results and discussion

The ISA-TAB-Nano specification makes use of the ISA-TAB Investigation, Study, and Assay files to record and share the assay metadata. In addition to sharing the assay metadata, we also want to share the endpoint measurements and description (chemical composition, characteristics) of both nanomaterials and small molecules (e.g., drugs, imaging agents). To accommodate this additional information, we have made extensions to ISA-TAB. In particular, the ISA-TAB Study file is suited for recording the source and characteristics of biological specimens and for specifying any treatments performed on them. However, the Study file is not designed to represent information about the chemical composition and characteristics of *material samples* (nanomaterials, drugs, imaging agents, and other small molecules). To represent this information, we have introduced a fourth ISA-TAB-Nano file called the Material file. We use the Material file to describe *material samples* while using the ISA-TAB Study file to describe *biospecimens*. We have modified the definitions of some of the field names in the ISA-TAB Investigation, Study, and Assay files and added new fields names to expand the scope of information that needs to be shared using the ISA-TAB-Nano specification. Detailed information of the field names and definitions of each file format can be found in Supporting Information. For more detailed information about the structure and contents of the ISA-TAB files, the reader may refer to the ISA-TAB specification [25]. The rules for naming the four file formats defined in the ISA-TAB-Nano specification are i_xxxx.txt for the Investigation file, s_xxxx.txt for the Study file, a_xxxx.txt for the Assay file, and m_xxxx.txt for the Material file, where ‘xxxx’ denotes a unique string.

The following sections describe the different components of the ISA-TAB-Nano files. Supporting Information provides additional materials to supplement the manuscript discussion, including a summary of extensions to the ISA-TAB format, the standard specification, and examples.

### Types of information shared using ISA-TAB-Nano files

The types of information shared using ISA-TAB-Nano pertain to the material samples, biospecimens, assays, and protocols. As listed in Table 1, we use the ISA-TAB-Nano Material file to capture information about material samples and references to the external material data files (e.g., image files, structural data files); the Investigation file to enter reference information about each investigation, study, assay, protocol: the Study file to capture information about biospecimen source and characteristics, names and attributes of protocols used in the preparation of samples analyzed in an assay; and, the Assay file to capture information about measured values of endpoint variables and references to external files (raw/derived data files, image files) related to each analyzed biospecimen or material sample. In the following sections, we explain how to record and share the different types of information in each ISA-TAB-Nano file.

**Table 1:** Types of information entered in the four ISA-TAB-Nano files

**No.**	**ISA-TAB-Nano file**	**Types of information entered in each ISA-TAB-Nano file**
1.	Investigation file	Reference information about each investigation, study, assay, protocol, Study file, and Assay file.
2.	Study file	Names and attributes of protocols used for preparing samples for analysis; source and characteristics of biospecimens.
3.	Assay file	Values of measured endpoint variables and references to external data files for each analyzed sample.
4.	Material file	Descriptions of the material sample and its structural parts and chemical components; linkage descriptions between chemical components; reference information about external material data files.

### Sharing material sample description

In the ISA-TAB-Nano framework, all material samples are viewed as formulations having one or more chemical components (e.g., nanoparticles, small molecules, solvent medium, etc.). The ISA-TAB-Nano material file has been designed for recording descriptions of various types of formulations, ranging from a simple chemical compound to a complex multi-component nanoparticle formulation.

The layout of the ISA-TAB-Nano Material file is same as that of the Study and Assay files, wherein the field names are arranged as columns and material sample information is entered as rows. Columns in the ISA-TAB-Nano Material file provide information on the material and its components, the relationship between materials and components, and any associated data files, as mentioned in Table 1.

### Using the ISA-TAB-Nano Material file to describe a material sample and its components

**Figure 1:**
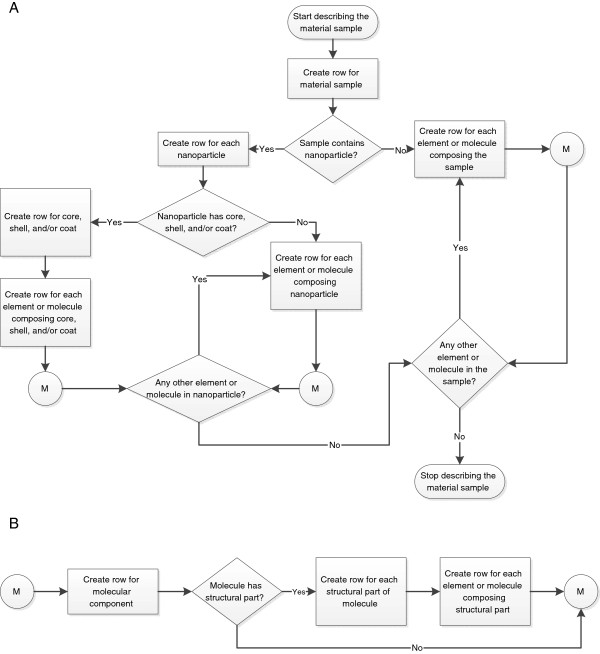
**Flowchart depicting the ways to identify different components of a material sample, and to guide the creation of rows for each component in the ISA-TAB-Nano Material file.** (**A**) The overall ISA-TAB-Nano file creation process which includes (**B**) the creation of molecular component entries.

The Material file is used to describe a material sample and its structural and chemical components. There are different ways to identify the components, depending on how the sample is viewed at its various levels of molecular and structural granularities. Figure 1 shows a process flowchart that can be used as a basis for identifying different components of the material sample. Once a component is identified, its description can be entered as a row in the Material file.

**Figure 2:**
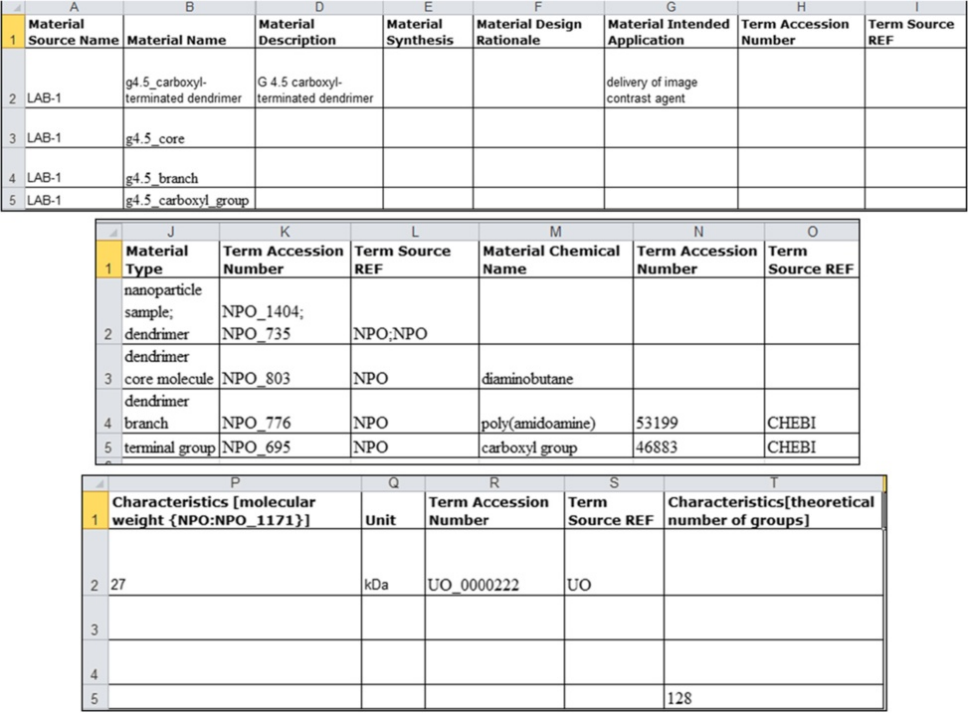
Example of an ISA-TAB-Nano Material file with information about a dendrimer nanoparticle sample, identified as LAB-1.

**Figure 3:**
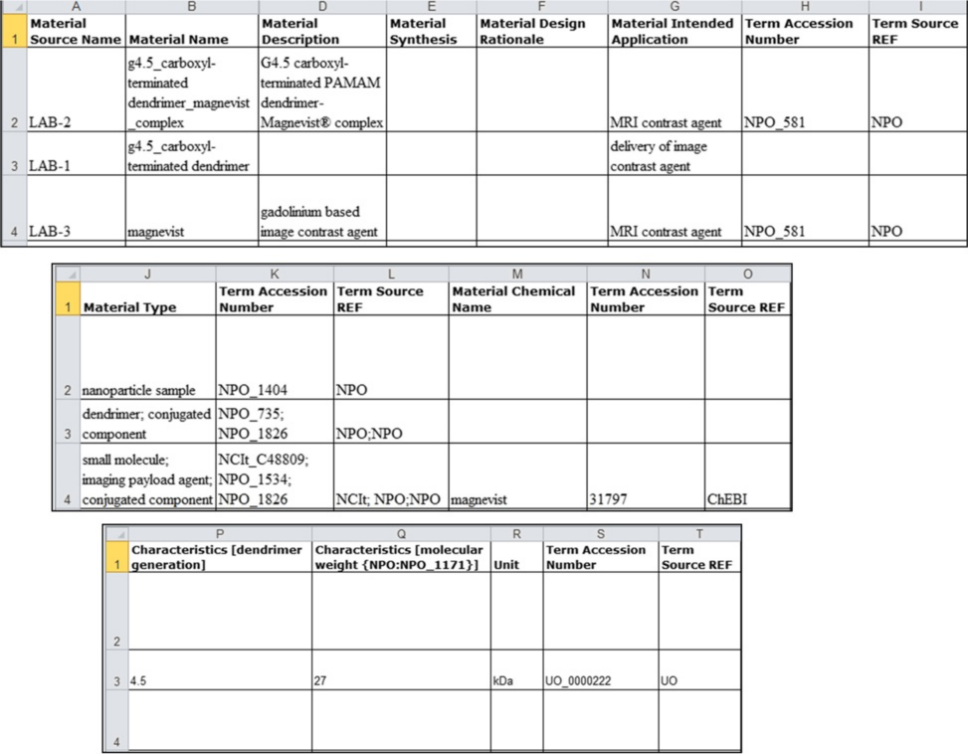
Example of an ISA-TAB-Nano Material file with information about a dendrimer-magnevist nanoparticle complex sample, identified as LAB-2.

In Figure 2, we show an example of the ISA-TAB-Nano Material file that contains information about a hypothetical dendrimer sample (LAB-1) and its components. The first row (row 2) of the Material file is used to identify the material sample name (identifier, chemical name), type, synthesis type, design rationale, intended application, and nominal characteristics. Subsequent rows of the Material file are used to identify the different structural parts and chemical components of the sample including their name, type, nominal characteristics, and intended application. While row 2 represents the dendrimer sample LAB-1, rows 3, 4, and 5 represent the components (core molecule, branch, and terminal group) of the dendrimer sample. From the given information, one can understand that the dendrimer sample (LAB-1) is a 4.5 generation dendrimer that has a molecular weight of 27 kilodaltons (kDa). Ontology terms can be identified by the name of the source ontology (defined by *Term Source REF* labels) and their identification numbers (defined by *Term Accession Number* labels) used within the ontology. Thus, we use row 2 to describe the material sample as a whole, and subsequent rows to describe each material sample component. If a material sample that is described in a Material file is also a component of another material sample, then the components of the former sample need not be specified in the Material file of the latter sample. To illustrate this scenario, we consider another hypothetical sample called LAB-2 that contains both LAB-1 and an image contrast agent called Magnevist^®^ (LAB-3). Figure 3 shows the LAB-2 Material file, where row 2 represents the whole LAB-2 material sample and rows 3 and 4 represent its components (LAB-1 and LAB-3). Note that we did not have to enter information about each component of LAB-1 since that information is already present in the LAB-1 Material file (see Figure 2). The LAB-2 Material file describes the material sample LAB-2 which consists of nanoparticles composed of carboxyl-terminated PAMAM dendrimer complexed with Magnevist^®^, a small molecule gadolinium-based image contrast agent. The dendrimer is conjugated to the Magnevist^®^ and is intended to be used as a delivery vehicle for image contrast agents like Magnevist^®^ in MRI applications.

### Using the ISA-TAB-Nano Material file to describe linkages in a material sample

**Figure 4:**
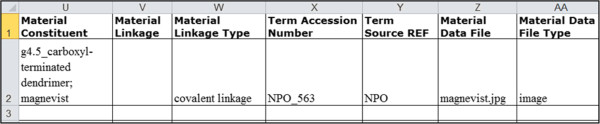
An example showing the representation of linkages among components of a nanoparticle sample in the ISA-TAB-Nano Material file.

The ISA-TAB-Nano Material file is used to describe the whole material sample and its components with respect to their name, type, characteristics, synthesis, design rationale, and intended application. In addition, the Material file can be used to identify the chemical components and represent how they are linked or connected to each other with respect to a nanoparticle. Any two chemical components in a typical multi-component nanoparticle formulation can be linked to each other by one of the three types of linkages: encapsulation, entrapment, or covalent linkage [28]. We can describe linkages between the different chemical components of a nanoparticle sample as shown in Figure 4. In the figure, one can identify that there is a covalent linkage that exists between the dendrimer and the Magnevist^®^ of LAB-2 sample. The term “covalent linkage” is used here as a generic term. Normally, if the specific type of covalent linkage is known (e.g., amide linkage, disulfide linkage, etc.), then one should use the appropriate term identifying that linkage. The NPO [28] provides several terms (their names and definitions) for describing different types of linkages that can be typically found in a nanoparticle sample. Additionally, we have recently developed a simple nomenclature system for describing the specific chemical linkages in nanoparticle formulations [30]. It is important to ensure that the names of the two chemical components specified in the *Material Constituent* field (Figure 4) match their respective names given in the *Material Name* field.

### Using the ISA-TAB-Nano Material file to record information about external files that provide additional information about the material sample

The Material file is also used to record the name or URI of external files that contain additional information about the material sample. Examples include image files displaying the chemical structures of molecules, or documents (e.g., spreadsheet, Word, PDF, etc.) that contain detailed descriptions of the sample. The example given in Figure 4 references one file called “magnevist.jpg” that contains an image of the molecular structure of Magnevist^®^.

In practice, one ISA-TAB-Nano Material file should be created for each material sample in a study. When using the format “m_xxxx.txt” to name the Material files, the string “xxxx” should exactly match the string used in the *Material Source Name* field of the sample’s Material file. For example, we would name the Material file of samples LAB-1 and LAB-2 as m_LAB-1.txt and m_LAB-2.txt, respectively.

### Sharing assay information

Different types of assays are performed to measure the physicochemical, *in vitro*, and *in vivo* properties of material samples. We can identify the type of each assay based on the measured endpoint, measurand, and technique used. For example, if an assay is related to a size characterization study, the endpoint could be “size” and the technique could be “dynamic light scattering (DLS)”, or “scanning electron microscopy (SEM)”, or other relevant technique. The corresponding assay types are “size by DLS assay”, “size by SEM”, etc. Assays of the same type may be performed using different protocols, by different people, at different dates, and under different experimental conditions. To interpret the characterization results of a material sample meaningfully, we need to have information about the assay type; biospecimen information (if it is a bioassay); protocol descriptions; dates of synthesis and analysis of the material sample; the measured endpoint variables; as well as the protocol variables of the experiment. Based on the ISA-TAB specification, variables that are kept constant in an assay experiment are termed “parameters”, while variables that are changed for studying their effects on the measured endpoint values are termed “study factors”.

### Representing physiochemical characterization data of material samples using ISA-TAB-Nano files

In a physicochemical characterization study, one measures the properties of a material sample and studies how these properties are affected by the study factors. Typically, one prepares several dilutions of same material sample and may even suspend each sample in different test media to achieve solution conditions suitable for performing a specific assay. Therefore, each analyzed sample may have its own identifier while its (source) material sample to be characterized has a different identifier (the *Material Source Name*).

**Figure 5:**
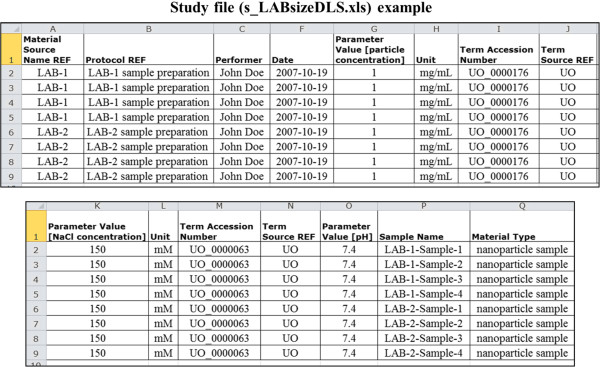
An example showing the layout of the ISA-TAB-Nano Study file.

**Figure 6:**
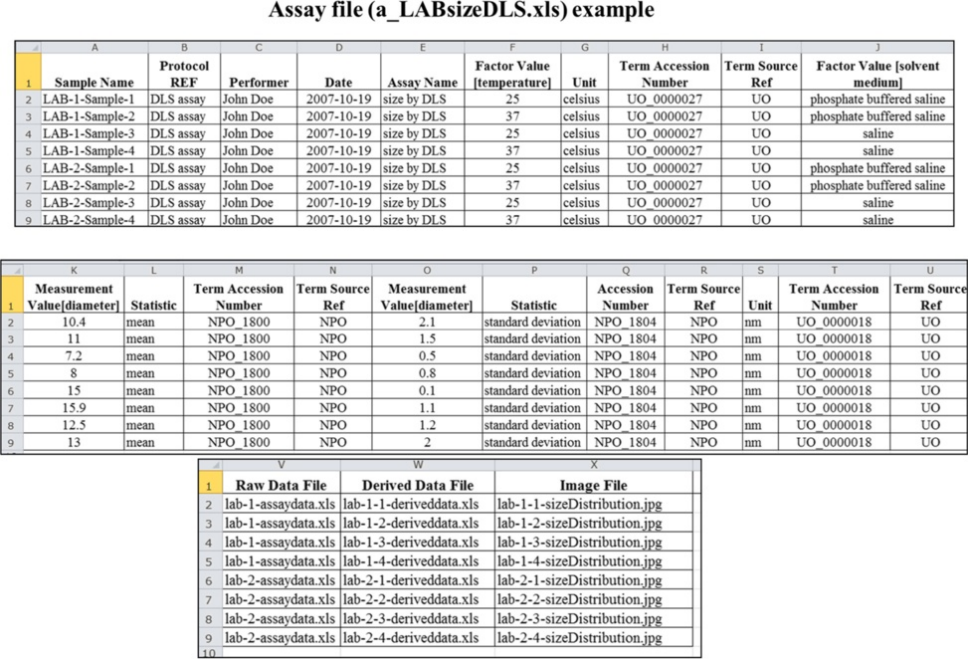
An example showing the layout of the ISA-TAB-Nano Assay file.

Figure 5 and Figure 6 show an example of how to use the Study and Assay files, respectively, to represent the assay data (metadata, summary data) of each sample analyzed in a size characterization assay using DLS technique. In this example, the goal of the study was to determine the effect of varying temperature and solvent medium on the size of nanoparticles in the two material samples - LAB-1 and LAB-2.

#### Study file

In the ISA-TAB Study file, a column *Source Name* is used to define the source of a biospecimen. ISA-TAB-Nano extends the ISA-TAB Study file by adding a column called *Material Source Name REF* to refer the source name of the material sample, given in the *Material Source Name* column of the sample’s Material file. Thus, as shown in Figure 5, the first column of the Study file (*Material Source Name REF*) represents the identifier of the material sample that is to be characterized. The second column contains the name of the protocol applied to prepare each individual biospecimen or material sample for the analysis. After the protocol column, we create columns to capture the attributes of the protocol including the name of the person who performed the protocol, the date when the protocol was applied, and the values of each parameter in the protocol. After these, we add two more columns to record the sample name (identifier) and its material type.

#### Assay file

The first column of the Assay file is used to enter the biospecimen or material sample names under the header name *Sample Name* (see Figure 6) – note that these names should match the names entered in the *Sample Name* column of the Study file. The names of the assay and protocol are entered in the next two columns. As in the Study file, we also create columns to capture the attributes of each protocol, including investigator, date, and parameter values. We also create columns for entering the values of each factor value and measurement value variables, followed by columns to record the URIs or names of associated data files, as shown in Figure 6.

The values for the parameters, factors, and measurements can be qualified by a unit of measurement and a statistical type (if applicable) using column headers *Unit* and *Statistic*, respectively, in both the Study as well as in the Assay files (see Figure 5 and Figure 6). Note that the *Measurement Value [<measurement name>]* and *Statistic* columns are newly added by the ISA-TAB-Nano specification. If terms from an ontology are used as valid values in any of the columns of the Study and Assay files, it is necessary to enter the term’s identifier and source ontology name in adjacent columns named as *Term Accession Number* and *Term Source REF*, respectively (not shown in the Figure 5 and Figure 6). One may choose to enter the factor values either in the Study file and/or in the Assay file, as appropriate to the actual experimental procedure(s). Furthermore, to enter any additional type of information in the Study or Assay file, one may create a column with header name, *Comment [<string>]*, as specified by ISA-TAB.

#### Investigation file

**Figure 7:**
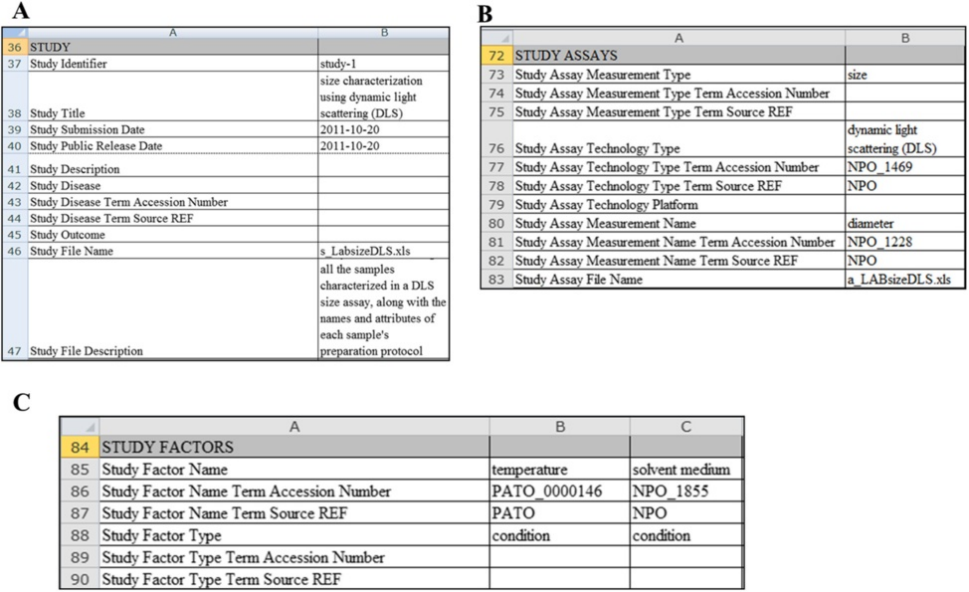
Examples showing how information is represented in the following sections of the Investigation file: (A) Study, (B) Study Assays, and (C) Study Factors.

**Figure 8:**
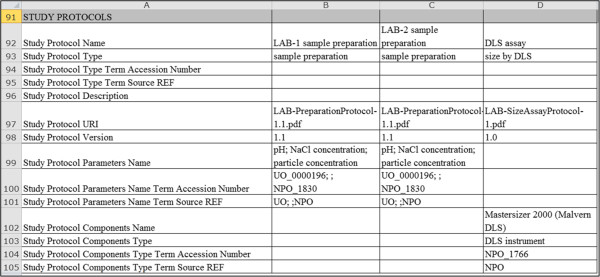
An example showing how information is represented in the Study Protocols section of the Investigation file.

The Investigation file is a reference for the different assays and protocols that have been conducted in a single study. The Investigation file is divided into several sections (listed in Table 2), including Study, Study Assays, Study Factors, and Study Protocols. As summarized in Table 2, the Study section is used to record information about each study and its Study file (see Figure 7A). The Study Assays section is used to record information about each assay and its assay file, particularly, the assay and technology types, measurement variable names, and the assay file names (see example in Figure 7B). The Study Factors section is used to record the names of factor variables and the factor type, as shown in Figure 7C. The Study Protocols section is used to record information about each protocol, including the name, type, description, URI file references and version of each protocol; as well as names of parameter variables and components (instruments, reagents, and software) of each protocol (an example is given in Figure 8). It should be noted the above information must be recorded in the Investigation file for reference to them in the individual Assay and Study files.

**Table 2:** Types of information entered in the eleven sections of the ISA-TAB-Nano Investigation file

**No.**	**Sections of Investigation file**	**Types of information entered in each section**
1.	Ontology Source Reference	Information about the ontologies referenced in the ISA-TAB-Nano files.
2.	Investigation	Investigation title and description.
3.	Investigation Contacts	Point(s) of contact for the whole investigation.
4.	Investigation Publications	References to publications of an investigation.
5.	Study	Information about each study and its associated Study file.
6.	Study Design Descriptors	Type of study design.
7.	Study Contacts	Point(s) of contact for a study.
8.	Study Publications	References to publications of a study.
9.	Study Assays	Information about each assay and its associated Assay file.
10.	Study Factors	Names of factor variables and types.
11.	Study Protocols	Information about each protocol.

Note that the ontologies or controlled vocabularies referenced in any of the ISA-TAB-Nano files must be declared in the Ontology Reference section of the Investigation file (noted in Table 2). The Investigation file can also contain information about publications and point of contact associated with an investigation or a specific study within the investigation (also noted in Table 2). For more details on the Investigation file, we refer the reader to the ISA-TAB specification [25] and ASTM ISA-TAB-Nano specification [31].

### Representing material sample *in vitro* and *in vivo* characterization data of material samples using ISA-TAB-Nano files

**Figure 9:**
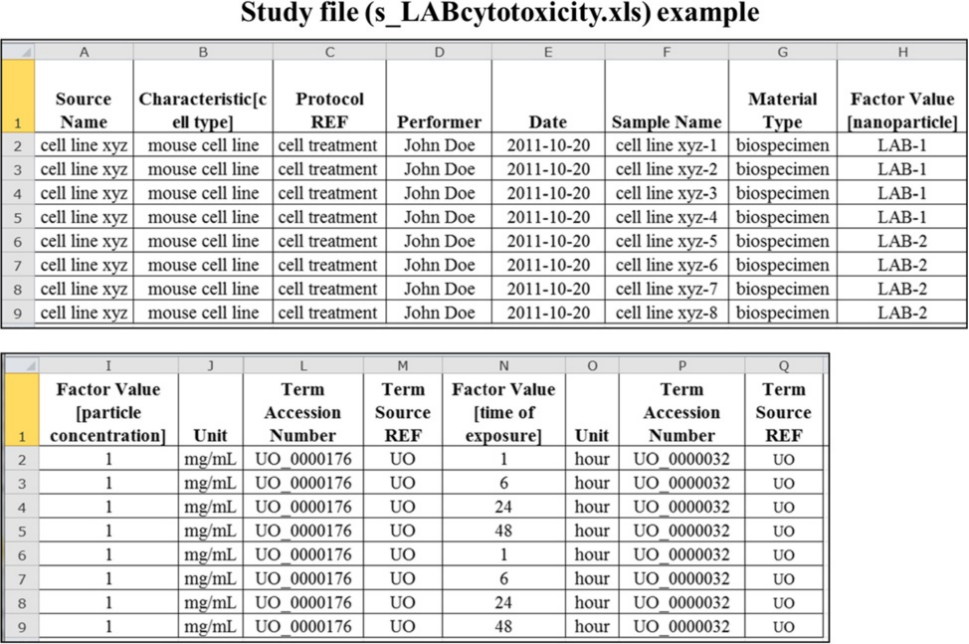
**A representative Study file for a hypothetical *in vitro *cytotoxicity characterization study, showing the description of a biological specimen and attributes of the sample preparation protocols.** Note that the entered information is not from an actual study and is only used for illustration purposes.

For *in vitro* and *in vivo* characterization studies, one is interested in measuring the effects of a material sample on a biological system, such as a cell line or animal model. In these studies, the material sample plays the role of a factor, and the sample analyzed is a biospecimen. The way we enter the *in vitro*/*in vivo* characterization data is similar to how we entered the physicochemical characterization data in the Investigation, Study, and Assay files. The only additional information to be entered is the description of the source and characteristics of the biospecimen in the Study file, as illustrated in Figure 9. Note that we use the column *Characteristic [<name>]* to enter the type of the biological source, as specified by ISA-TAB. The material type of the sample is entered as biospecimen in the *Material Type* column, and the material representing the nanoparticle or other non-biological material is entered in the *Factor Value* column. Particularly, the non-biological material entered in the *Factor Value* column should be the same as that entered in the *Material Source Name* column of the sample’s Material file.

### Integrated view of ISA-TAB-Nano files and external referenced documents

**Figure 10:**
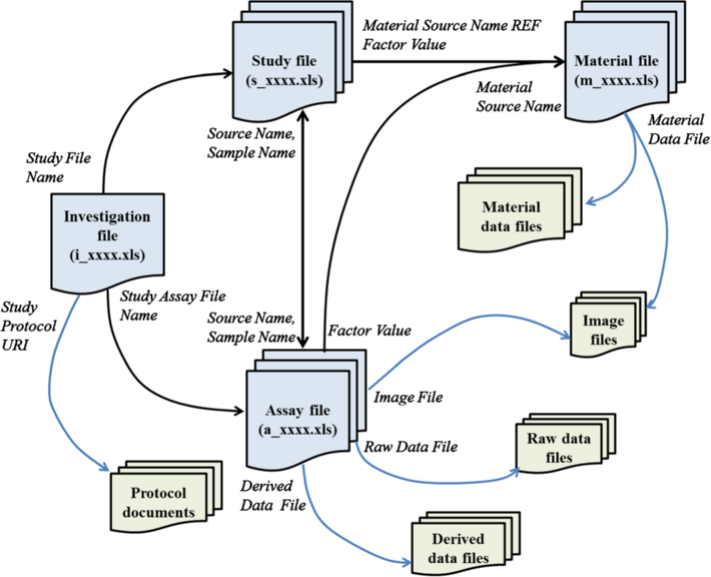
Links (black arrows) between ISA-TAB-Nano files and references (blue arrows) to documents (protocol documents, raw/derived data files, image files, and material description files).

ISA-TAB-Nano files support the aggregation of all information pertaining to the description and characterization of material samples. In Figure 10, we show how the ISA-TAB-Nano files and the external files, such as protocol documents, raw data files, derived data files, image files, and material sample description files, can be assembled within the ISA-TAB-Nano framework. The names of all Study and Assay files are recorded in the Investigation file in columns corresponding to the fields *Study File Name* (Study section) and *Study Assay File Name* (Study Assays section), respectively. As a result, the Investigation, Study and Assay files are linked. The data in the Study and Assay files for each sample are integrated through the *Sample Name* fields found in both of the files. This is illustrated in Figure 5 and Figure 6, where the sample names in Column A of the assay file (a_LABsizeDLS.xls) are the same as those found in Column P of the study file (s_LABsizeDLS.xls). Thus, the information of each biospecimen or material sample in the Study and Assay files can be linked to each other. If the sample that is analyzed is a material sample, then it is necessary to ensure that the value of *Material Source Name REF* entered in the Study file matches the string entered in the *Material Source Name* of its Material file (Figure 10). Similarly, the same string must be used in the *Factor Value* column of the study file or assay file for material samples that are used as factors in an *in vitro* or an *in vivo* study. As shown in Figure 10, the ISA-TAB-Nano files can also be associated with any external files, as long as the URI or the names of these files are referenced in the appropriate fields in the ISA-TAB-Nano files. Specifically, the ISA-TAB-Nano files are associated with protocol documents by referencing the URIs or names of these documents in the *Study Protocol URI* field of the Investigation file. The external files related to the description of a material sample are associated via the *Material Data File* field of the material file. Finally, the Assay file associates the raw data, derived data and image files via the column fields, *Raw Data File*, *Derived Data File*, and *Image File*, respectively.

### Validation of ISA-TAB-Nano files

A key feature of ISA-TAB-Nano (and the parent ISA-TAB format) is that it can be readily created and edited with any spreadsheet software, without the need for specialized bioinformatics support. However, this also creates challenges because such software does not enforce validation rules for ISA-TAB-Nano files. One possible solution to this problem is to adapt the suite of open source tools provided by the ISA-TAB community [16-18], such as: (1) ISACreator, which provides a graphical user interface for entering in data into ISA-TAB files; (2) ISAValidator, which assesses the validity of ISA-TAB files; (3) ISAConverter, which converts an ISA-TAB file to one of several other formats if so desired, (4) ISAConfigurator, which creates the XML files that define specialized versions of ISA-TAB, and (5) bioinvindex, which is a database for storing ISA-TAB files. These tools are part of our future plans to be tested for editing and validating ISA-TAB-Nano files.

XML-based validation is also of interest and would involve developing an ISA-TAB-Nano XML schema based on the ISA-TAB-Nano specification such that any ISA-TAB-Nano XML file converted from the ISA-TAB-Nano files can be formally validated against the XML schema. Having an XML-based validation service would also enable software systems to import and export validated ISA-TAB-Nano files to and from distributed nanotechnology resources.

## Conclusions

In this paper, we have provided an overview of ISA-TAB-Nano, a spreadsheet-based exchange format for information pertaining to the description and characterization of nanomaterials. The ISA-TAB-Nano specification extends the ISA-TAB specification that was designed for sharing all types of ‘omics data. Specifically, ISA-TAB-Nano leverages the three primary ISA-TAB file formats - Investigation file, the Study file, and the Assay file - to represent the metadata related to the characterization of nanomaterials. To capture the measurement data, ISA-TAB-Nano specifies new fields in the Investigation file and in the Assay file. In addition, ISA-TAB-Nano includes a new file format, the Material file, to capture the description of nanomaterials and chemical substances (drugs, imaging agents, etc.) that are characterized in an assay. The ISA-TAB-Nano specification was recently submitted as an ASTM work item to obtain community feedback and to promote ISA-TAB-Nano as a voluntary consensus standard for the nanotechnology community. Additionally, we hope that the ISA-TAB-Nano standard will be adopted by journals and other organizations to ensure a uniform representation of information that is too often “lost” in a prose Methods section and cannot be easily extracted for re-use.

Many of the ISA-TAB extensions developed for ISA-TAB-Nano could also be used for studies of other types of complex materials or formulations. In particular, the “Material” file addition is likely to be useful outside of the nanotechnology realm for a broader range of samples with complex composition or structural characteristics.

Key to promoting the successful adoption of ISA-TAB-Nano will be the availability of resources to facilitate the creation and validation of these files. Our adherence to the parent ISA-TAB specification will allow the ready adaptation of the ISA Tools [16-18] to support the extended framework, an anticipated next step for this project. The ability easily select ontology terms when populating the files is critical to the ultimate goal of integrating these data across disparate resources. The ISA-TAB and ISA-TAB-Nano specifications support the use of any ontology or controlled vocabulary; in this domain, preferred ontologies include NanoParticle Ontology (NPO) [28], Chemical Entities of Biological Interest (ChEBI) [32], Gene Ontology (GO) [33], NCI thesaurus (NCIt) [34], and Unit Ontology (UO) [35]. The Nano WG continues to expand the NanoParticle Ontology (NPO) to support the needs of ISA-TAB-Nano, and is considering various methods and tools for facilitating the use of ontology terms in ISA-TAB-Nano files. (Additional files 1, 2 and 3).

## Competing interests

The authors have no competing interests to declare.

## Authors’ contributions

All authors contributed to the design and testing of the ISA-TAB-Nano standard. DGT, SG, SLH, JDK, and NAB were primarily responsible for the preparation of the manuscript with contributions and revisions from the other authors. All authors read and approved the final manuscript.

## Supplementary Material

Additional file 1 This Supporting Information provides a summary of ISA-TAB-Nano extensions to the ISA-TAB format, glossaries for the terms used in the ISA-TAB-Nano specification, and an overview of the worked examples and templates provided in the other supporting files. Click here for file

Additional file 2 This file contains the examples described in Supporting File 1. Click here for file

Additional file 3 This file contains ISA-TAB-Nano templates for common assays. Click here for file
