# Supplementary material for: ISA-TAB-Nano: A Specification for Sharing Nanomaterial Research Data in Spreadsheet-based Format
Source: BMC Biotechnol. 2013 Jan 14;13:2. doi: 10.1186/1472-6750-13-2 (PMC3598649; doi:10.1186/1472-6750-13-2)
Supplement: Additional file 1 — This Supporting Information provides a summary of ISA-TAB-Nano extensions to the ISA-TAB format, glossaries for the terms used in the ISA-TAB-Nano specification, and an overview of the worked examples and templates provided in the other supporting files. [file 1472-6750-13-2-S1.doc]

# ISA-TAB-Nano: A Specification for Sharing Nanomaterial Research Data in Spreadsheet-based Format

Supporting information

Dennis G Thomas[[1]](#footnote-2), Sharon Gaheen[[2]](#footnote-3), Stacey L. Harper[[3]](#footnote-4), Martin FrittsError: Reference source not found, Fred Klaessig[[4]](#footnote-5), Elizabeth Hahn-Dantona[[5]](#footnote-6), David Paik[[6]](#footnote-7), Sue PanError: Reference source not found, Grace A. Stafford[[7]](#footnote-8), Elaine T. Freund[[8]](#footnote-9), Juli D. Klemm[[9]](#footnote-10), Nathan A. Baker[[10]](#footnote-11)

Contents

[Summary of extensions to the ISA-TAB format 1](#__RefHeading___Toc338329217)

[Glossaries 3](#__RefHeading___Toc338329218)

[Assay file term glossary 3](#__RefHeading___Toc338329219)

[Investigation file term glossary 4](#__RefHeading___Toc338329220)

[Material file term glossary 12](#__RefHeading___Toc338329221)

[Study file term glossary 15](#__RefHeading___Toc338329222)

[Overview of examples and templates 17](#__RefHeading___Toc338329223)

# Summary of extensions to the ISA-TAB format

| **ISA-TAB Extension Field Name** | **ISA-TAB File** | **Purpose** |
| --- | --- | --- |
| Investigation Disease | Investigation | To capture and retrieve investigations associated with specific disease modalities such as cancer |
| Investigation Disease Term Accession Number | Investigation | To enable semantic interoperability for disease terms |
| Investigation Disease Term Source REF | Investigation | To enable semantic interoperability for disease terms |
| Investigation Outcome | Investigation | To enable researchers to review the outcomes of investigations for assessing the utility of the investigation in achieving scientific endpoints |
| Study Disease | Investigation | To capture and retrieve studies associated with specific disease modalities such as cancer |
| Study Disease Term Accession Number | Investigation | To enable semantic interoperability for disease terms |
| Study Disease Term Source REF | Investigation | To enable semantic interoperability for disease terms |
| Study Outcome | Investigation | To enable researchers to review the outcomes of studies for assessing the utility of the investigation in achieving scientific endpoints |
| Study Factor Name Term Accession Number | Investigation | To enable semantic interoperability for study factor name |
| Study Factor Name Term Source REF | Investigation | To enable semantic interoperability for study factor names |
| Study Assay Measurement Name | Investigation | To capture the variables measured in an assay to support cross study analysis |
| Study Assay Measurement Name Term Accession Number | Investigation | To enable semantic interoperability for assay measurement names |
| Study Assay Measurement Name Term Source REF | Investigation | To enable semantic interoperability for assay measurement names |
| Material Source Name REF | Study | To provide a reference for the material associated with the study and identfied in the Material file |
| Material File | Study | To provide a reference for the ISA-TAB-Nano material file that describes the material samples of a study |
| Measurement Value | Assay | To record the endpoint value of an assay measurement within the assay file |
| Statistic | Assay | To capture the statistical measures (e.g. mean, SD) associated with a measurement or factor value |
| All Fields | Material | To describe nanomaterials and small molecules |

# Glossaries

## Assay file term glossary

| **Term** | **Definition** |
| --- | --- |
| Sample Name | The unique identification name of the sample, which is referred to from within the Study file.  Sample Name is a node in the Assay file, and it links the Assay file to the Study file. This concept is taken from ISA-TAB. |
| Source Name | The unique identification name of the source referred to from within the Study file. This column is used only if the source is same as the sample in an assay.  The Source Name links the Assay file to the Study file. This concept is taken from ISA-TAB. |
| Protocol REF | The name of the protocol used to perform the experiment. This name should be obtained from a value for the field “Study Protocol Name” in the investigation file (i.e., within the study protocols section).  This concept is adapted from ISA-TAB. |
| ParameterValue[parameter term] | Value of a parameter, which is kept constant, when applying a protocol. The parameter term is written within brackets and must match the term used as value for the Study Protocol Parameter Name in the ISA-TAB-Nano Investigation File.  This concept is taken from ISA-TAB. |
| Performer | The name of the person who carried out the protocol. This concept is taken from ISA-TAB. |
| Date | The calendar day on which the protocol was carried out. The date format should be in YYYY-MM-DD.  This concept is taken from ISA-TAB. |
| Assay Name | The name of the assay performed. The name is used as an identifier within the Assay file. Qualifying headers for Assay Name are Performer, Date, and Comment[]. This concept is taken from ISA-TAB. |
| Unit | The standard of measurement used if the values in Characteristic[], Parameter Value[] or Factor Value [] columns are quantitative and dimensional.  This concept is adapted from ISA-TAB. |
| Term Accession Number | Identification number of a term selected from an ontology or a controlled vocabulary, if the term is entered as a value in Source Name, Material Type, Characteristic [], Parameter Value[], Unit or Factor Value[] columns. This concept is taken from ISA-TAB. |
| Term Source Ref | The name which identifies the source from where a term is selected and entered in ISA-TAB-Nano study files. This name should match one of the names entered in the Term Source Name field. This concept is taken from ISA-TAB. |
| Factor Value[factor term] | The value of an independent variable manipulated by the experimentalist with the intention to affect the subject of study (i.e. stressor). Factor terms are described in brackets (Syntax: Factor Value[<factor term>]). Factor Value[] in Assay file should reference technical variations (such as software, instrument or protocol variations). This concept is adapted from ISA-TAB. |
| Comment | Any comment that provides additional information, which is added only when no other appropriate field exists.  This concept is taken from ISA-TAB. |
| Measurement Value[measurement term] | The endpoint of the assay. Measurement value terms are described in brackets (Syntax: Measurement Value[<measurement term>]).  This concept is introduced in ISA-TAB-Nano to capture measurement outputs recorded in summary data. |
| Statistic | The type of statistical measure attributed to a numerical value (e.g., mean, standard deviation, z-average, etc.). This is a required field, if the parameter value, factor value, or measurement value is a statistical measure. |
| Image File | The name or URI of an image file generated from an assay. This concept is taken from ISA-TAB. |
| Derived Data File | The name or URI of the file resulting from data transformation or processing.  This concept is taken from ISA-TAB. |
| Raw Data File | The name or URI of the raw data files. This concept is taken from ISA-TAB. |

## Investigation file term glossary

| ONTOLOGY SOURCE REFERENCE | Section header for the ontology source reference section. This section is used to record information about ontologies containing the terms referenced in the ISA-TAB-Nano files. |
| --- | --- |
| Term Source Name | Name of the source of a term. The source could be an ontology or a controlled vocabulary. The source name is the full name or the acronym of the ontology/controlled vocabulary. This is a required field if the term source name is referenced in any of the ISA-TAB-Nano files.  This concept is taken from ISA-TAB. |
| Term Source File | A file name or a URI of the official source named in the “Term Source Name" field. This concept is taken from ISA-TAB. |
| Term Source Version | Version number of the source file that contains the term This is a required field if the field for “Term Source File” has a value.  This concept is taken from ISA-TAB. |
| Term Source Description | Text description to disambiguate resources when homologous acronyms are used. This concept is taken from ISA-TAB. |
| INVESTIGATION | Section header for the investigation section. This section allows for the description of the investigation. |
| Investigation Identifier | A locally unique identifier or an accession number provided by a repository. This concept is taken from ISA-TAB. |
| Investigation Title | A concise phrase used as a title for the investigation. This concept is adapted from ISA-TAB. |
| Investigation Description | A textual description of the investigation. This concept is taken from ISA-TAB. |
| Investigation Disease | Disease(s) that are the subject of the investigation, if applicable. This concept is introduced in ISA-TAB-Nano. |
| Investigation Disease Term Accession Number | Identification number of a term selected from an ontology or a controlled vocabulary, if the term is entered as a value for Investigation Disease. This concept is introduced in ISA-TAB-Nano. |
| Investigation Disease Term Source REF | The name which identifies the source from where the term for Investigation Disease is selected. This name should match one of the names entered in the Term Source Name field. This concept is introduced in ISA-TAB-Nano. |
| Investigation Outcome | A textual description of the outcome(s) of an investigation. This concept is introduced in ISA-TAB-Nano. |
| Investigation Submission Date | The date on which the investigation was reported to a repository (format: YYYY-MM-DD). This concept is taken from ISA-TAB. |
| Investigation Public Release Date | The date on which the investigation is publicly released or published (format: YYYY-MM-DD). This concept is taken from ISA-TAB. |
| INVESTIGATION CONTACTS | Section header for the Investigation Contacts section. This section allows for the identification of the point(s) of contact for the investigation. |
| Investigation Person Last Name | The last name of a person who is the point of contact for the investigation. This concept is taken from ISA-TAB. |
| Investigation Person First Name | The first name of a person who is the point of contact for the investigation. This concept is taken from ISA-TAB. |
| Investigation Person Mid Initials | The middle initial(s) of a person who is the point of contact for the investigation. This concept is a modification of the concept "Investigation Person Mid Initials" used in ISA-TAB. |
| Investigation Person Email | The email address of a person who is the point of contact for the investigation. This concept is taken from ISA-TAB. |
| Investigation Person Phone | The telephone number of the point of contact for the investigation. This concept is taken from ISA-TAB. |
| Investigation Person Fax | The fax number of a person who is the point of contact for the investigation. This concept is taken from ISA-TAB. |
| Investigation Person Address | The mailing address of a person who is the point of contact for the investigation. This concept is taken from ISA-TAB. |
| Investigation Person Affiliation | The name of the organization to which the point of contact belongs. This concept is taken from ISA-TAB. |
| Investigation Person Roles | The term which classifies the role(s) performed by person who is the point of contact for the investigation. This concept is taken from ISA-TAB. |
| Investigation Person Roles Term Accession Number | Identification number of a term selected from an ontology or a controlled vocabulary, if the term is entered as a value for Investigation Person Role. This concept is taken from ISA-TAB. |
| Investigation Person Roles Term Source REF | Name of the ontology or controlled vocabulary from which a term is selected and entered as a value for Investigation Person Role. This concept is taken from ISA-TAB. |
| INVESTIGATION PUBLICATIONS | Section header for the Investigation Publications section. This section allows for the identification of articles (published) associated with the investigation. |
| Investigation PubMed ID | PubMed Identifier of the publication associated with the investigation.  This concept is taken from ISA-TAB. |
| Investigation Publication DOI | A Digital Object Identifier (DOI) of the publication associated with the investigation. This concept is taken from ISA-TAB. |
| Investigation Publication Author list | A semicolon-delimited (";") list of authors of a publication associated with the investigation.  This concept is adapted from ISA-TAB. |
| Investigation Publication Title | A concise phrase used as a title for the publication associated with the investigation.  This concept is taken from ISA-TAB. |
| Investigation Publication Status | A term describing the status of a publication (i.e., submitted, in preparation, published). This concept is taken from ISA-TAB. |
| Investigation Publication Status Term Accession Number | The identification number of a term selected from an ontology or a controlled vocabulary, if the term is entered as a value for Investigation Publication Status. This concept is taken from IS-TAB. |
| Investigation Publication Status Term Source REF | The name which identifies the source from where the term for Investigation Publication Status is selected. This name should match one of the names entered in the Term Source Name field. This concept is taken from ISA-TAB. |
| STUDY | Section header for the Study section. This section allows for the description of one or more studies conducted as part of an investigation. |
| Study Identifier | A unique identifier used for the study. It is either a temporary identifier supplied by users or one generated by a repository or other database.  This concept is taken from ISA-TAB. |
| Study Title | A concise phrase used to encapsulate the purpose and goal of the study. This concept is taken from ISA-TAB. |
| Study Submission Date | The date on which the study is submitted to an archive (format: YYYY-MM-DD). This concept is taken from ISA-TAB. |
| Study Public Release Date | The date on which the study is publicly released or published (format: YYYY-MM-DD).  This concept is taken from ISA-TAB. |
| Study Description | A textual description of the study, with components such as objectives or goals. This concept is taken from ISA-TAB. |
| Study Disease | Disease(s) that are the subject of the study, if applicable.  This concept is introduced in ISA-TAB-Nano. |
| Study Disease Term Accession Number | Identification number of a term selected from an ontology or a controlled vocabulary, if the term is entered as a value for Study Disease. This concept is introduced in ISA-TAB-Nano. |
| Study Disease Term Source REF | The name which identifies the source from where the term for Study Disease is selected. This name should match one of the names entered in the Term Source Name field. This concept is introduced in ISA-TAB-Nano. |
| Study Outcome | A textual description about the outcome(s) of the study. This concept is introduced in ISA-TAB-Nano. |
| Study File Name | The name of the ISA-TAB-Nano study file, which lists information about the biological specimens (cells, tissues, organs, animal model, body fluids), nanoparticles, small organic molecules, and other types of samples assayed in a study. There can be only one file per cell. This concept is taken from ISA-TAB. |
| Study File Description | A textual description which provides additional information on the ISA-TAB-Nano study file. This concept is introduced in ISA-TAB-Nano. |
| STUDY DESIGN DESCRIPTORS | Section header for the study design descriptors section. This section allows for the identification of design type of the study. |
| Study Design Type | A term describing the classification of the study based on the overall study (experimental) design (e.g. comparison study). The term can be free-text or taken from a controlled vocabulary/ontology. This concept is taken from ISA-TAB. |
| Study Design Type Term Accession Number | Identification number of a term selected from an ontology or a controlled vocabulary, if the term is entered as a value for Study Design Type.  This concept is taken from ISA-TAB. |
| Study Design Type Term Source REF | The name which identifies the source from where the term for Study Design Type is selected. This name should match one of the names entered in the Term Source Name field. This concept is taken from ISA-TAB. |
| STUDY CONTACTS | Section header for the Study Contacts section. This section allows for the identification of the point of contact for a study. |
| Study Person Last Name | The last name of a person who is the point of contact for the study. This concept is taken from ISA-TAB. |
| Study Person First Name | The first name of a person who is the point of contact for the study. This concept is taken from ISA-TAB. |
| Study Person Mid Initials | The middle initial(s) of a person who is the point of contact for the study. This concept is a modification of the concept "Study Person Mid Initials" used in ISA-TAB. |
| Study Person Email | The email address of a person who is the point of contact for the study. This concept is taken from ISA-TAB. |
| Study Person Phone | The telephone number of the point of contact for the study. This concept is taken from ISA-TAB. |
| Study Person Fax | The fax number of a person who is the point of contact for the study. This concept is taken from ISA-TAB. |
| Study Person Address | The mailing address of a person who is the point of contact for the study. This concept is taken from ISA-TAB. |
| Study Person Affiliation | The name of the organization to which the point of contact belongs. This concept is taken from ISA-TAB. |
| Study Person Roles | The term which classify the role(s) performed by person who is the point of contact for the study. This concept is taken from ISA-TAB. |
| Study Person Roles Term Accession Number | Identification number of a term selected from an ontology or a controlled vocabulary, if the term is entered as a value for Study Person Role.  This concept is taken from ISA-TAB. |
| Study Person Roles Term Source REF | The name which identifies the source from where the term for Study Person Role is selected.  This name should match one of the names entered in the Term Source Name field.  This concept is taken from ISA-TAB. |
| STUDY PUBLICATIONS | Section header for the publication section of a study. This section allows for the identification of articles (published) associated with the study. |
| Study PubMed ID | PubMed Identifier of the publication associated with the study.  This concept is taken from ISA-TAB. |
| Study Publication DOI | A Digital Object Identifier (DOI) of the publication associated with the study. This concept is taken from ISA-TAB. |
| Study Publication Author list | A semicolon-delimited (";") list of authors of a publication associated with the study.  This concept is adapted from ISA-TAB. |
| Study Publication Title | A concise phrase used as a title for the publication associated with the study.  This concept is taken from ISA-TAB. |
| Study Publication Status | A term describing the status of a publication (i.e., submitted, in preparation, published), associated with the study. This concept is taken from ISA-TAB. |
| Study Publication Status Term Accession Number | The identification number of a term selected from an ontology or a controlled vocabulary, if the term is entered as a value for Study Publication Status. This concept is taken from ISA-TAB. |
| Study Publication Status Term Source REF | The name which identifies the source from where the term for Study Publication Status is selected. This name should match one of the names entered in the Term Source Name field. This concept is taken from ISA-TAB. |
| STUDY ASSAYS | Section header for the Study Assay section. This section allows for the identification of type of measurement and the type of technology used for the measurement in an assay that is part of a study. |
| Study Assay Measurement Type | A term to qualify the endpoint, or what is being measured. This concept is taken from ISA-TAB. |
| Study Assay Measurement Type Term Accession Number | Identification number of the term selected from an ontology or a controlled vocabulary, if the term is entered as a value for Study Assay Measurement Type. This concept is taken from ISA-TAB. |
| Study Assay Measurement Type Term Source REF | The name which identifies the source from where the term for Study Assay Measurement Type is selected. This name should match one of the names entered in the Term Source Name field. This concept is taken from ISA-TAB. |
| Study Assay Technology Type | The type of technology (technique or method) used for the assay measurement (e.g., Dynamic light scattering).  This concept is taken from ISA-TAB. |
| Study Assay Technology Type Term Accession Number | Identification number of a term selected from an ontology or a controlled vocabulary, if the term is entered as a value for Study Assay Technology Type. This concept is taken from ISA-TAB. |
| Study Assay Technology Type Term Source REF | The name which identifies the source from where the term for Study Assay Technology Type is selected. This name should match one of the names entered in the Term Source Name field. This concept is taken from ISA-TAB. |
| Study Assay Technology Platform | The manufacturer and platform name of the instruments used in the study assay. This concept is taken from ISA-TAB. |
| Study Assay Measurement Name | A semicolon-delimited list of names of quantities whose values are the outputs of an assay measurement. This concept is introduced in ISA-TAB-Nano. |
| Study Assay Measurement Name Term Accession Number | Identification number of a term selected from an ontology or a controlled vocabulary, if the term is entered as a value for Study Assay Measurement Name. This concept is introduced in ISA-TAB-Nano. |
| Study Assay Measurement Name Term Source REF | The name which identifies the source from where the term for Study Assay Measurement Name is selected. This name should match one of the names entered in the Term Source Name field. This concept is introduced in ISA-TAB-Nano. |
| Study Assay File Name | Name of the ISA-TAB-Nano Assay file corresponding to the study assay.  This is a required field. There can be only one assay file name per cell. This concept is taken from ISA-TAB. |
| STUDY FACTORS | Section header for the Study Factor section. This section allows for the identification of factors associated with the study. |
| Study Factor Name | The name of one independent variable (factor) manipulated by the experimentalist with the intention to affect the subject of study (i.e. Stressor).  This is a required field, if there are factors in an assay study. Only one factor name is allowed per cell. The value of a factor is given either in the Study or in the Assay file. This concept is adapted from ISA-TAB. |
| Study Factor Name Term Accession Number | Identification number of the term selected from an ontology or a controlled vocabulary, if the term is entered as a value for Study Factor Name. This concept is taken from ISA-TAB. |
| Study Factor Name Term Source REF | The name which identifies the source from where the term for Study Factor Name is selected. This name should match one of the names entered in the Term Source Name field. This concept is taken from ISA-TAB. |
| Study Factor Type | A term used for the classification of factors associated with the study (e.g. condition). This is a required field if there are factors in an assay study. More than one term is allowed per cell; multiple terms are separated by semicolons. This concept is adapted from ISA-TAB |
| Study Factor Type Term Accession Number | Identification number of a term selected from an ontology or a controlled vocabulary, if the term is entered as a value for Study Factor Type.  This concept is taken from ISA-TAB. |
| Study Factor Type Term Source REF | The name which identifies the source from where the term for Study Factor Type is selected. This name should match one of the names entered in the Term Source Name field. This concept is taken from ISA-TAB. |
| STUDY PROTOCOLS | Section header for the Study Protocols section. This section allows for the identification of type of protocols, and the parameters and components of a protocol used in a study. A protocol describes the formal plan of an experiment or research activity, including the objective, rationale, design, materials and methods for the conduct of the study; intervention description, and method of data analysis. |
| Study Protocol Name | The name of the protocols used within a study and will be referenced in the ISA-TAB-Nano Study and Assay files.  This concept is taken from ISA-TAB. |
| Study Protocol Type | The term used to classify the protocol (e.g., synthesis, assay, etc.). This is a required field. This concept is taken from ISA-TAB. |
| Study Protocol Type Term Accession Number | Identification number of a term selected from an ontology or a controlled vocabulary, if the term is entered as a value for Study Protocol Type. This concept is taken from ISA-TAB. |
| Study Protocol Type Term Source REF | Name of the ontology or controlled vocabulary from which a term is selected and entered as a value for Study Protocol Type.This concept is taken from ISA-TAB. |
| Study Protocol Description | A textual description of the protocol. This concept is taken from ISA-TAB. |
| Study Protocol URI | Pointer to protocol documents or resources external to ISA-TAB-Nano, which can be accessed by their Uniform Resource Identifier (URI). If URI is not available, enter the protocol document file name and make the protocol document available along with ISA-TAB-Nano files.  This concept is adapted from ISA-TAB. |
| Study Protocol Version | An identifier for the version to ensure protocol tracking.  This concept is taken from ISA-TAB. |
| Study Protocol Parameters Name | A semicolon-delimited (";") list of parameter names used as an identifier in the ISA-TAB-Nano Study or Assay files. A protocol parameter is a constant associated with a protocol, which is not varied as part of an experiment. This concept is taken from ISA-TAB. |
| Study Protocol Parameters Name Term Accession Number | Identification number of a term selected from an ontology or a controlled vocabulary, if the term is entered as a value for the Study Protocol Parameters Name. This concept is taken from ISA-TAB. |
| Study Protocol Parameters Name Term Source REF | The name which identifies the source from where the term for Study Protocol Parameter Name is selected. This name should match one of the names entered in the Term Source Name field. This concept is taken from ISA-TAB. |
| Study Protocol Components Name | A semicolon-delimited (";") list of names identifying the components of a protocol. Component names include instrument names, software names, and reagent names.  This concept is taken from ISA-TAB. |
| Study Protocol Components Type | The term to classify the protocol component, e.g., instrument, software, and reagent. This concept is taken from ISA-TAB. |
| Study Protocol Components Type Term Accession Number | Identification number of a term selected from an ontology or a controlled vocabulary, if the term is entered as a value for the Study Protocol Components Type. This concept is taken from ISA-TAB. |
| Study Protocol Components Type Term Source REF | The name which identifies the source from where the term for Study Protocol Component Type is selected. This name should match one of the names entered in the Term Source Name field. This concept is taken from ISA-TAB. |

## Material file term glossary

| **Term** | **Definition** |
| --- | --- |
| Material Source Name | The unique identification name of the source from which the material sample is derived. Its value is used as the value for “Source Name” in ISA-TAB-Nano Study files, thereby linking the ISA-TAB-Nano Material file and the study file.  This concept is introduced in ISA-TAB-Nano. |
| Material Name | The unique identification name for the sample and its different components. This concept is introduced in ISA-TAB-Nano. |
| Manufacturer Lot Identifier | A distinctive numeric, alpha, or alpha-numeric identification code assigned by the manufacturer or distributor. It is assigned to a specific quantity of manufactured material or product that is produced in a manner that is expected to render it homogeneous.This concept is introduced in ISA-TAB-Nano. |
| Material Description | A textual description of the material sample. This concept is introduced in ISA-TAB-Nano. |
| Material Synthesis | A text or a single term description of how the material was made. This concept is introduced in ISA-TAB-Nano. |
| Material Design Rationale | A text description for the underlying design rationale is the property, process or phenomenon taken into consideration when formulating a nanoparticle or other substance in order to achieve the intended use of the formulation. This concept is introduced in ISA-TAB-Nano. |
| Material Intended Application | The application for which a drug, nanoparticle or other substance is formulated and tested (e.g., MRI). This concept is introduced in ISA-TAB-Nano. |
| Term Accession Number | Identification number of a term selected from an ontology or a controlled vocabulary, if the term is entered as a value for Material Intended Application. This concept is introduced in ISA-TAB-Nano. |
| Term Source REF | The name which identifies the source from where the term for Material Intended Application is selected. This name should match one of the names entered in the Term Source Name field. This concept is introduced in ISA-TAB-Nano. |
| Material Type | One or more terms used to classify the type of material sample. Multiple terms are entered as a semicolon-delimited list.  This concept is introduced in ISA-TAB-Nano. |
| Term Accession Number | Identification number of a term selected from an ontology or a controlled vocabulary, if the term is entered as a value for Material Type. This concept is introduced in ISA-TAB-Nano. |
| Term Source REF | The name which identifies the source from where the term for Material Type is selected. This name should match one of the names entered in the Term Source Name field. This concept is introduced in ISA-TAB-Nano. |
| Material Chemical Name | The chemical name of the material or its constituent material. This concept is introduced in ISA-TAB-Nano. |
| Term Accession Number | Identification number of a term selected from an ontology or a controlled vocabulary, if the term is entered as a value for Material Chemical Name. This concept is introduced in ISA-TAB-Nano. |
| Term Source REF | The name which identifies the source from where the term for Material Chemical Name is selected. This name should match one of the names entered in the Term Source Name field. This concept is introduced in ISA-TAB-Nano. |
| Characteristics | A semicolon-delimited list of terms that describe specific attributes of the sample or its constituents.  This concept is introduced in ISA-TAB-Nano. |
| Term Accession Number | Identification number of a term selected from an ontology or a controlled vocabulary, if the term is entered as a value for Material Characteristic. This concept is introduced in ISA-TAB-Nano. |
| Term Source REF | The name which identifies the source from where the term for Material Characteristic is selected. This name should match one of the names entered in the Term Source Name field. This concept is introduced in ISA-TAB-Nano. |
| Characteristics Value | The value corresponding to a material characteristic name. For example, “single walled carbon nanotube” is the value of the characteristic name “wall type” for the material chemical name “carbon nanotube”.  This concept is introduced in ISA-TAB-Nano. |
| Term Accession Number | Identification number of a term selected from an ontology or a controlled vocabulary, if the term is entered as a value for Material Characteristic Value. This concept is introduced in ISA-TAB-Nano. |
| Term Source REF | The name which identifies the source from where the term for Material Characteristic Value is selected. This name should match one of the names entered in the Term Source Name field. This concept is introduced in ISA-TAB-Nano. |
| Unit | The standard of measurement used if the value for Material Characteristic Value is quantitative and dimensional.  This concept is introduced in ISA-TAB-Nano. |
| Term Accession Number | Identification number of a term selected from an ontology or a controlled vocabulary, if the term is entered as a value for Material Characteristic Unit. This concept is introduced in ISA-TAB-Nano. |
| Term Source REF | The name which identifies the source from where the term for Material Characteristic Unit is selected. This name should match one of the names entered in the Term Source Name field. This concept is introduced in ISA-TAB-Nano. |
| Material Constituent | The material name for each of the components of the nanoparticle sample. The names should be obtained from the entries for the “Material Name” field.  This concept is introduced in ISA-TAB-Nano. |
| Material Linkage | A unique identification name for each of the two components (whole or part) that are linked to each other in the nanoparticle sample. The names should be obtained from the entries for the “Material Name” field. This is a required field, if the field “Material Linkage Type” is not empty. A cell must have the names of the two linked components, separated by a semicolon. This concept is introduced in ISA-TAB-Nano. |
| Material Linkage Type | The type of linkage present in a nanoparticle sample (e.g., attachment, encapsulation, entrapment etc.).  This concept is introduced in ISA-TAB-Nano. |
| Term Accession Number | Identification number of a term selected from an ontology or a controlled vocabulary, if the term is entered as a value for Material Linkage Type. This concept is introduced in ISA-TAB-Nano. |
| Term Source REF | The name which identifies the source from where the term for Material Linkage Type is selected. This name should match one of the names entered in the Term Source Name field. This concept is introduced in ISA-TAB-Nano. |
| Material Data File | The name of files (e.g. Image, Structures file) containing information about the material sample.  There can be only one file name per cell. This concept is introduced in ISA-TAB-Nano. |
| Material Data File Type | The name that defines the type of the material file (e.g. image, graph).  This concept is introduced in ISA-TAB-Nano. |
| Term Accession Number | Identification number of a term selected from an ontology or a controlled vocabulary, if the term is entered as a value for Material Data File Type.This concept is introduced in ISA-TAB-Nano. |
| Term Source REF | The name which identifies the source from where the term for Material Data File Type is selected. This name should match one of the names entered in the Term Source Name field. This concept is introduced in ISA-TAB-Nano. |
| Material Data File Version | The version number (e.g. 1.0) of the material file.  This concept is introduced in ISA-TAB-Nano. |
| Material Data File Description | A textual description providing additional information on the material file. This concept is introduced in ISA-TAB-Nano. |

## Study file term glossary

| **Term** | **Definition** |
| --- | --- |
| Source Name | The unique identification name of the biological source from where the sample is derived. Source names can be qualified using the following column headers: Source Name is a node in the Study file. Characteristic[], Material Type, Material File, and Comment. If the sample assayed is a biological specimen, its source is the starting biological material from which the sample was derived after the application of a protocol. The name of the source typically refers to the cell line or animal number for biological specimens. This concept is taken from ISA-TAB. |
| Material Source Name REF | The reference to the material source name specified in the Material file. The material source name should match the value recorded for Material Source Name in the corresponding ISA-TAB-Nano Material file. The material source name identifies the source from where the material sample is derived. If the sample assayed is a nanoparticle sample or some chemical substance not derived from a biological material, then the corresponding material source name should refer to the starting sample that was modified by a protocol for the assay. The material source name column should only be used for physical-chemical or other characterizations in which the material is the primary sample. For in vitro and in vivo characterizations in which the biological specimen is the primary sample, the material should be referenced as a material factor (e.g. Factor Value [material]). This concept was introduced in ISA-TAB-Nano. |
| Sample Name | The unique identification name of the sample. The sample is obtained after the application of a protocol. Sample Name is a node in the Study file. Sample names can be qualified using the following column headers: Characteristic[], Material Type, Material File, Provider, and Comment. This concept is adapted from ISA-TAB. |
| Material Type | An attribute for the sample type (e.g., biospecimen, nanoparticle sample, small molecule etc.). The term can be a free-text description or taken from an ontology or a controlled vocabulary. If it is the latter, then the following qualifiers are used: Term Accession Number and Term Source REF. This concept is taken from ISA-TAB. |
| Material File | The name of the ISA-TAB-Nano Material file that contains detailed descriptions of the source samples. Note: This column header is not applicable for biospecimens. This concept is introduced in ISA-TAB-Nano. |
| Characteristics[ ] | An attribute for Source Name and Sample Name. Characteristic terms are written within brackets (e.g., Characteristic [organism], Characteristic [cell type], etc.). This concept is taken from ISA-TAB. |
| Provider | An attribute for Source Name, which refers to the name of the person or the vendor providing the source sample. This concept is adapted from ISA-TAB. |
| Protocol REF | The name of the protocol used to prepare the sample. This name should match a value for the field “Study Protocol Name” in the investigation file (within the study protocols section). This concept is taken from ISA-TAB. |
| Parameter Value[parameter term] | Value of a parameter, which is kept constant, when applying a protocol. The parameter term is written within brackets and must match the term used as value for the Study Protocol Parameter Name in the ISA-TAB-Nano Investigation File. This concept is taken from ISA-TAB. |
| Performer | The name of the person who carried out the protocol. This concept is taken from ISA-TAB. |
| Date | The calendar day on which the protocol was carried out. The date format should be in YYYY-MM-DD. If there are other dates to be recorded (e.g., the date when a sample was received from a vendor), one should create a Comment[] column and specify the type of date within the square brackets (e.g., Comment[date received]). This concept is adapted from ISA-TAB. |
| Unit | The standard of measurement used if the values in Characteristic[], Parameter Value[] or Factor Value [] columns are quantitative and dimensional. This concept is adapted from ISA-TAB. |
| Term Accession Number | Identification number of a term selected from an ontology or a controlled vocabulary, if the term is entered as a value in Source Name, Material Type, Characteristic [], Parameter Value[], Unit or Factor Value[] columns. This concept is taken from ISA-TAB. |
| Term Source Ref | The name which identifies the source from where a term is selected and entered in ISA-TAB-Nano study files. This name should match one of the names entered in the Term Source Name field. This concept is taken from ISA-TAB. |
| Factor Value[factor term] | The value of an independent variable manipulated by the experimentalist with the intention to affect the subject of study (i.e. stressor). Factor terms are given in brackets, and must be defined in the ISA-TAB-Nano investigation file in the STUDY FACTORS section. This concept is adapted from ISA-TAB. |
| Comment | Any comment that provides additional information, which is added only when no other appropriate field exists. This concept is taken from ISA-TAB. |

# Overview of examples and templates

The table below identifies the supporting information for the ISA-TAB-Nano specification.

| **Folder / File** | **Description** |
| --- | --- |
| **examples** | The examples folder contains example ISA-TAB-Nano files including an Investigation File and associated Study, Material, and Assay files. The example provided is from an investigation performed by the Nanotechnology Characterization Laboratory (NCL):  **Investigation Title:** Nanotechnology Characterization Laboratory (NCL) Dendrimer-Based MRI Contrast Agent   **Investigation Description:** The objective of this investigation is to characterize a PAMAM dendrimer with an associated gadolinium chelate MRI contrast agent. NCL studies addressed in this report can be divided into three main categories: physicochemical characterization; immunotoxicology; in vitro toxicology. |
| examples/i_NCL2006A.xls | The Investigation file for the NCL Dendrimer-Based MRI contrast agent investigation. |
| examples/m_NCL-20.xls  examples/m_NCL-21.xls  examples/m_NCL-22.xls  examples/m_NCL-23.xls  examples/m_NCL-24.xls  examples/m_NCL-25.xls  examples/m_NCL-26.xls | The Material files for the NCL Dendrimer-Based MRI contrast agent investigation. The nanomaterials submitted for testing at the NCL were: NCL-20, G4 tris (hydroxyl) terminated PAMAM dendrimer; NCL-21, G4 pyrrolidinone terminated PAMAM dendrimer; NCL-22, G4.5 COONa terminated PAMAM dendrimer; NCL-23, G4.5 COONa terminated PAMAM dendrimer-Magnevist® complex; NCL-25, G4 tris (hydroxyl) terminated PAMAM dendrimer-Magnevist® complex; and NCL-26, G4 pyrrolidinone terminated PAMAM dendrimer-Magnevist® complex. Commercially available Magnevist® (NCL-24) was used as a control. |
| examples/s_size-DLS.xls | The Study file for the NCL size by Dynamic Light Scattering (DLS) study. |
| examples/s_cytoxicity-LLCPK1.xls | The Study file for the NCL Hep G2 Hepatocarcinoma Cytotoxicity Study involving lactate dehydrogenase (LDH) release |
| examples/a_size-DLS.xls | The Assay file for the NCL size by DLS assay. |
| examples/a_LDK-LLCPK1.xls | The Assay file for the NCL [Hep G2 Hepatocarcinoma Cytotoxicity Assayhttps://ncisvn.nci.nih.gov/svn/isa-tab-nano/trunk/examples/ncl/a_LDH-LLCPK1.xls](https://ncisvn.nci.nih.gov/svn/isa-tab-nano/trunk/examples/ncl/a_LDH-LLCPK1.xls) LDH assay. |
| **templates** | The templates folder provides a blank template (no date) for each ISA-TAB-Nano file including an Investigation File and associated Study, Material, and Assay files. Users can populate the template files with user specific data sets. |
| templates/i_xxx.xls | The template Investigation file |
| templates/m_xxx.xls | The template Material file |
| templates/s_xxx.xls | The template Study file |
| templates/a_xxx.xls | The template Assay file |

1. Knowledge Discovery and Informatics, Pacific Northwest National Laboratory, Richland, WA 99352, USA [↑](#footnote-ref-2)
2. Health Solutions, Science Applications International Corporation (SAIC) , Rockville, MD 20852, USA [↑](#footnote-ref-3)
3. Department of Environmental and Molecular Toxicology, School of Chemical, Biological and Environmental Engineering, Oregon State University, Corvallis, OR 97331, USA [↑](#footnote-ref-4)
4. Pennsylvania Bio Nano Systems, LLC, Doylestown, PA, USA [↑](#footnote-ref-5)
5. Lockheed Martin, Rockville, MD 20852, USA [↑](#footnote-ref-6)
6. Department of Radiology, Stanford University, Stanford, CA 94305, USA [↑](#footnote-ref-7)
7. The Jackson Laboratory, Bar Harbor, Maine 04609, USA [↑](#footnote-ref-8)
8. 3^rd^ Millennium, Inc., North Smithfield, RI 02896, USA [↑](#footnote-ref-9)
9. Center for Biomedical Informatics and Information Technology, National Cancer Institute, Rockville, MD 20852, USA [↑](#footnote-ref-10)
10. To whom correspondence should be addressed. Knowledge Discovery and Informatics, Pacific Northwest National Laboratory. PO Box 999, MSID K7-28, Richland, WA 99352. Phone: 509-375-3997, E-mail: [nathan.baker@pnnl.gov](mailto:nathan.baker@pnnl.gov) . [↑](#footnote-ref-11)
